# Association between dietary flavonoid intake and hypertension among U.S. adults

**DOI:** 10.3389/fimmu.2024.1380493

**Published:** 2024-04-03

**Authors:** Yingying Wan, Dan Ma, Qinghua Shang, Hao Xu

**Affiliations:** ^1^ National Clinical Research Center for Chinese Medicine Cardiology, Xiyuan Hospital, China Academy of Chinese Medical Sciences, Beijing, China; ^2^ China Academy of Chinese Medical Sciences, Xiyuan Hospital Suzhou Hospital, Suzhou, China

**Keywords:** flavonoid, anthocyanin, flavan-3-ol, hypertension, NHANES

## Abstract

**Background:**

Hypertension is one of the major risk factors for cardiovascular disease. Dietary flavonoids have been reported to reduce inflammation, protect against oxidative stress, protect the vascular endothelium, and improve vascular health. However, the relationship between dietary flavonoid intake and the prevalence of hypertension remains controversial.

**Methods:**

This study included 8010 adults from the 2007-2010 and 2017-2018 National Health and Nutrition Examination Surveys (NHANES). The relationship between dietary flavonoid intake and the prevalence of hypertension was explored by weighted logistic regression and weighted restricted cubic spline.

**Results:**

We found an inverse relationship between total anthocyanin intake and the prevalence of hypertension in the fourth quartile compared with the first quartile [0.81(0.66,0.99), p = 0.04]. Moreover, the prevalence of hypertension tended to decrease with increasing total anthocyanin intake in participants over 60 years of age. In addition, we found a U-shaped relationship between the prevalence of hypertension and total flavan-3-ol intake. Total flavan-3-ol intake was inversely associated with hypertension prevalence in the third quartile compared with the first quartile [0.79 (0.63,0.99), p = 0.04]. Moreover, there was a significant negative association between the prevalence of hypertension and total flavan-3-ol intake when total flavan-3-ol intake was below 48.26 mg/day.

**Conclusion:**

Our study found a negative association between the prevalence of hypertension and moderate total anthocyanins intake and total flavan-3-ols intake. Our study provides evidence from a population-based study for a negative association between dietary flavonoid intake and the prevalence of hypertension.

## 1 Introduction

Hypertension is a common chronic disease and hypertension is one of the major risk factors for cardiovascular disease (1, 2). Hypertension puts enormous pressure on healthcare and economic systems. From 1975 to 2015, the prevalence of hypertension worldwide increased from 594 million to 1.13 billion (3). 46% of US adults have hypertension in 2017 (4). Management of hypertension includes both pharmacologic and non-pharmacologic interventions. Non-pharmacologic interventions help to reduce the use of anti-hypertensive medications and slow the progression of pre-hypertension (5, 6).

Inflammation may be an important mechanism in the development of hypertension (7, 8). Inflammation is the body’s defense mechanism in the face of a pathogen attack. Excessive reactive oxygen/reactive nitrogen species produced during oxidative metabolism can activate an inflammatory response leading to the synthesis and secretion of pro-inflammatory cytokines (9). Oxidative stress and inflammation cause endothelial dysfunction and arterial damage, resulting in hypertension (10, 11). Studies have found that nutritional control can reduce inflammation (12).

Flavonoids are biologically active polyphenolic compounds of plant origin (13). Flavonoids can be categorized into six main subclasses based on their chemical structure, including anthocyanins, flavan-3-ols, flavanones, flavonoids, flavonols, and isoflavones (14). Flavonoid compounds have anti-oxidative stress, anti-inflammatory, anti-viral, cardioprotective, anti-diabetic, anti-cancer, and other effects (15, 16). Regular consumption of flavonoids is beneficial in reducing the risk of many chronic diseases such as cancer, cardiovascular diseases, and neurodegenerative diseases (17, 18). Flavonoid compounds were found to inhibit the NF-κB p65 and MAPK pathways and reduce inflammation (19, 20). Flavonoid compounds can improve endothelial function by activating vascular Akt and eNOS. Akt is involved in the proliferation of vascular endothelial cells, and NO is produced after activation of Enos (21). Increased NO bioavailability improves vasodilatation and circulation and protects the vascular endothelium by affecting protein kinases, ion channels, and phosphodiesterases and counteracting vascular inflammation and low-density lipoprotein oxidative stress (22). Intake of flavonoids can help improve vascular health and reduce the risk of developing hypertension and cardiovascular disease (23).

There is less evidence from previous population-based studies, with some cross-sectional studies finding an association between blood pressure reduction and anthocyanins and total flavan-3-ols (24, 25), but some studies have reported nonsignificant results (26–28).

Therefore, this study used all publicly available data from the USDA Food and Beverage Flavonoid Values Database Survey (referred to as the Flavonoid Database) for 2007-2010 and 2017-2018, as well as flavonoid intake data from NHANES, to explore the association between dietary flavonoid intake and the prevalence of hypertension in U.S. adults over the age of 20. It is hoped that our study will inform the relationship between dietary flavonoid intake and the prevalence of hypertension.

## 2 Materials and methods

### 2.1 Study population

Data for this study were collected from the NHANES. The NHANES database is a major epidemiologic survey program conducted by the National Center for Health Surveys (NCHS) of the U.S. Department of Health and Human Services (HHS) to assess the health and nutritional status of the U.S. population’s health and nutritional status. NHANES is released every 2 years and collects health and nutritional information about participants through sampling of representative samples, including physiologic measurements, health questionnaires, laboratory tests, and nutritional surveys. Specific information can be retrieved on the NHANES website (https://www.cdc.gov/nchs/nhanes/index.htm). The NCHS Ethics Review Board approved the NHANES study protocol, and each participant signed an informed consent form.

We collected 29,940 participants from the NHANES database for two consecutive NHANES cycles in 2007-2010 and 2017-2018. After excluding 6397 participants with missing data on hypertension, 5113 with missing flavonoid intake, 4572 aged less than 20 years, and 5848 with missing data on other variables, 8010 participants were finally included.

### 2.2 Assessment of flavonoid intakes

We collected data on dietary flavonoid intake from the Flavonoid Database. The Flavonoid Database provides the amounts (mg/100 g) of 29 flavonoids from 6 flavonoids in all foods/beverages in the USDA Food and Nutritional Database for Dietary Studies (FNDDS). Specific information can be obtained from the FNDDS website (https://www.ars.usda.gov/northeast-area/beltsville-md-bhnrc/beltsville-human-nutrition-research-center/food-surveys-research-group/docs/fndds-flavonoid-database/). The flavonoid values from The Flavonoid Database can be used for dietary data in the What We Eat in America (WWEIA) and the NHANES. In this study, dietary flavonoid intake data were collected from the Flavonoid Database for 2007-2010 and 2017-2018. We defined dietary flavonoid intake as the average of the two-day intake of each flavonoid.

### 2.3 Assessment of hypertension

Based on the questionnaire and physical examination results, participants were diagnosed with hypertension if they met one of the following 3 conditions (1): the average systolic blood pressure ≥130 mmHg or the average diastolic blood pressure ≥80 mmHg (2); the answer to the question “have you ever been told to take a prescription for hypertension” was “yes” (3); the answer to the question “have you ever been told that you had high blood pressure” was “yes”. All Blood pressure determinations (systolic and diastolic) were taken in the mobile examination center. Average blood pressure was calculated by the following protocol: The diastolic reading with zero was not used to calculate the diastolic average. If all diastolic readings were zero, then the average would be zero. If only one blood pressure reading was obtained, that reading is the average. If there was more than one blood pressure reading, the first reading was always excluded from the average.

### 2.4 Assessment of covariates

Our study included the following covariates: age, gender, race, education, smoking status, alcohol drinking, poverty-to-income ratio (PIR), BMI (body mass index is calculated as weight in kilograms divided by height in meters squared), daily energy intake, total metabolic equivalents of weekly physical activity (total MET of PA), and BMI (body mass index) is calculated as weight in kilograms divided by height in meters squared. Disease covariates include hyperlipidemia, congestive heart failure, stroke, heart attack, coronary heart disease, and diabetes.

Races include Non-Hispanic White, Non-Hispanic Black, Mexican American or Other. Education level includes less than 9th grade, less than 9th grade, 9-11th grade (includes 12th grade with no diploma), high school graduate/GED or equivalent, some college or AA degree, and college graduate or above. The family income-poverty ratio was classified as<1.5, 1.5-3.5, > 3.5. Smoking status was classified as never (smoked less than 100 cigarettes in life), former (smoked more than 100 cigarettes in life and smoke not at all now), now (smoked more than 100 cigarettes in life and smoke some days or every day). Alcohol drinking was classified as never (had <12 drinks in lifetime); former (had ≥12 drinks in 1 year and did not drink last year, or did not drink last year but drank ≥12 drinks in lifetime); Mild (defined as 2 drinks per day for men and 1 drinks per day for women); moderate (defined as 3 drinks per day for men and 2 drinks per day for women, or binge drinking 2-4 days per day); heavy (defined as ≥4 drinks per day for men and ≥3 drinks per day for women, or binge drinking ≥ 5 days per day).

Hyperlipidemia was identified when any of the subsequent criteria were met: triglycerides≧150 mg/dL; total cholesterol≧200 mg/dL; low-density lipoprotein≧130 mg/dL; high-density lipoprotein ≤ 40mg/dL (male); high-density lipoprotein ≤ 50mg/dL (female); or utilization of antihyperlipidemic agents. The diagnostic criteria for congestive heart failure: the answer to the question “Have you ever been told that you had congestive heart failure” was “yes”. The diagnostic criteria for heart attack: the answer to the question “Have you ever been told that you had a heart attack” was “yes”. The diagnostic criteria for stroke: the answer to the question “Have you ever been told that you had a stroke” was “yes”. The diagnostic criteria for coronary heart disease: the answer to the question “Have you ever been told that you had coronary heart disease” was “yes”. The diagnostic criteria for diabetes were: the doctor told you to have diabetes, HbA1c ≥ 6.5%; fasting glucose ≥ 7.0 mmol/L; random blood glucose ≥ 11.1 mmol/L; two-hour OGTT blood glucose ≥ 11.1 mmol/L; utilization of diabetes medication or insulin. DM: diabetes mellitus; IFG: Impaired Fasting Glycaemia (fasting glucose 6.1-7.0 mmol/L); IGT: Impaired Glucose Tolerance (two-hour OGTT blood glucose 7.8-11.1 mmol/L).

### 2.5 Statistical analysis

We used R software (version 4.1.3) for statistical analysis. We used the R software packages “NHANESR” and “survey” for data organization and statistical analysis. Continuous variables in the analysis of baseline information were expressed as weighted mean ± standard deviation and compared between groups using one-way ANOVA. Categorical variables were expressed as frequencies and percentages and were compared using chi-square tests. Four weighted logistic regression models were used to examine the relationship between flavonoid intake and prevalence of hypertension. The crude model was unadjusted. Model 1 was adjusted by age, race, sex, and BMI. Model 2 was adjusted by age, race, sex, BMI, daily energy intake, smoking status, alcohol drinking, and education. Model 3 was adjusted by age, race, sex, BMI, daily energy intake, smoking status, alcohol drinking, education, Total MET of PA, PIR, hyperlipidemia, heart attack, stroke, congestive heart failure, coronary heart disease, and diabetes. Weighted RCS from the “rms” package was used to assess potential nonlinear associations. Subgroup-weighted logistic regression was used to analyze the effect of flavonoid intake on the prevalence of hyperlipidemia. Weighted logistic regression was used to calculate odds ratios (OR) and corresponding 95% confidence intervals (CI). The critical value for statistical significance was p<0.05. P-values for interactions based on the log-likelihood ratio test were used to assess the heterogeneity of the relationship between subgroups.

## 3 Results

### 3.1 Characteristics of participants

**Figure 1:**
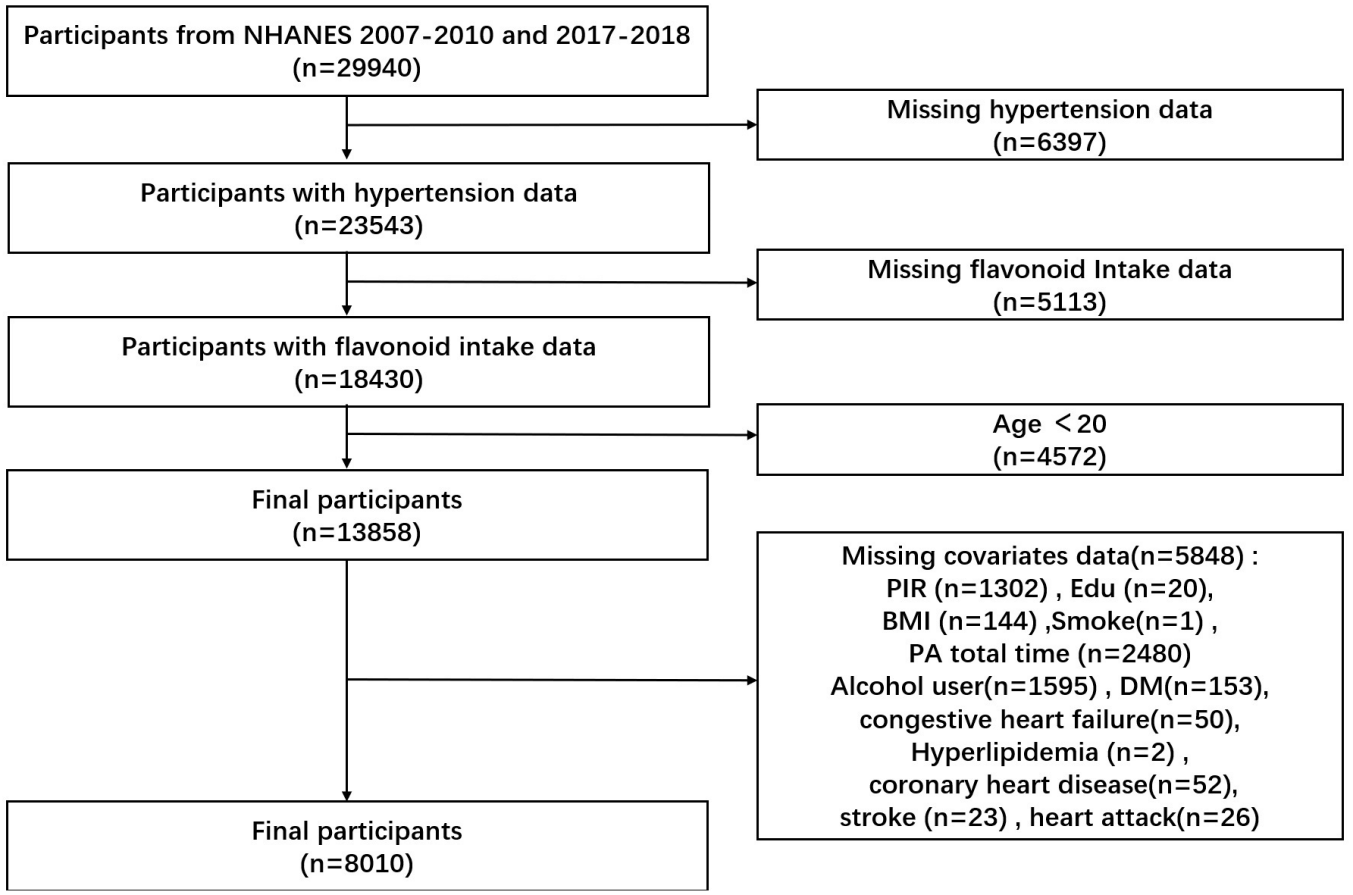
Flow chart of study participants.

This study included 8010 NHANES participants (**Figure 1**), representing 126.2 million noninstitutionalized residents of the United States. Of these 8010 participants, 2996 had hypertension with a mean age of 41.14 years and 5014 did not have hypertension with a mean age of 55.87 years (**Table 1**). Participants with hypertension were 54.22% male, higher than those without hypertension. In terms of race, participants with hypertension were more likely to be non-Hispanic white and non-Hispanic black. Participants who were college graduates or over were less likely to have hypertension. In terms of smoking status, participants who had ever smoked were more likely to have hypertension. Participants who had former consumed alcohol, mildly consumed alcohol, or never consumed alcohol were more likely to have hypertension. Participants with hypertension were not significantly different from healthy participants at PIR. Participants with hypertension had a mean BMI of 30.88 kg/m^2^, which was higher than those without hypertension. The mean daily energy intake of participants with hypertension was 2085.19 kcal, which was less than that of participants without hypertension. Participants with hypertension had less total MET of PA than participants without hypertension. Participants with hypertension were more likely to have a combination of congestive heart failure, hyperlipidemia, heart attack, coronary heart disease, and diabetes. Participants with hypertension had greater total Isoflavones intake, total flavan-3-ols intake, total flavonols intake, and total sum of all 29 flavonoid intake compared to participants without hypertension.

**Table 1:** Characteristics of participants in the study.

Variable	Participants without hypertension (n=5014)	Participants with hypertension (n=2996)	*P-*value
**Age (years)**	41.14(0.33)	55.87(0.42)	< 0.0001
Sex, N (%)			0.01
Female	2482(50.76)	1379(45.78)	
Male	2532(49.24)	1617(54.22)	
Race, N (%)			< 0.0001
Non-Hispanic White	2442(70.95)	1520(73.55)	
Non-Hispanic Black	787(8.72)	698(11.58)	
Mexican American	856(8.43)	323(4.87)	
Others	929(11.91)	455(10.00)	
Education, N (%)			< 0.0001
Less than 9th grade	346(3.00)	262(4.17)	
9-11th grade (includes 12th grade with no diploma)	638(8.87)	393(9.10)	
High School graduate/GED or equivalent	1096(22.92)	748(26.87)	
Some college or AA degree	1559(30.01)	926(33.77)	
College graduate or above	1375(35.20)	667(26.10)	
Smoking status, N (%)			< 0.0001
Now	1144(20.50)	512(16.05)	
Former	1004(20.92)	979(32.53)	
Never	2866(58.58)	1505(51.41)	
Alcohol drinking, N (%)			< 0.0001
Former	512(8.02)	479(12.34)	
Heavy	1276(25.03)	491(17.98)	
Moderate	933(19.67)	435(16.80)	
Mild	1762(38.87)	1222(43.43)	
Never	531(8.41)	369(9.45)	
PIR, N (%)			0.64
<1.5	1664(21.92)	971(21.71)	
>3.5	1743(48.65)	1029(47.65)	
1.5-3.5	1607(29.43)	996(30.63)	
**BMI (kg/m2)**	27.73(0.18)	30.88(0.20)	< 0.0001
**Daily energy intake (kcal)**	2174.97(17.93)	2085.19(18.28)	< 0.001
**Total MET of PA (/week)**	5464.29(182.84)	4116.55(150.86)	< 0.0001
Congestive heart failure, N (%)			< 0.0001
No	4985(99.52)	2860(96.55)	
Yes	29(0.48)	136(3.45)	
Hyperlipidemia, N (%)			< 0.0001
No	1849(37.84)	565(18.75)	
Yes	3165(62.16)	2431(81.25)	
Stroke, N (%)			< 0.0001
No	4968(99.10)	2806(94.95)	
Yes	46(0.90)	190(5.05)	
Heart attack, N (%)			< 0.0001
No	4942(98.86)	2798(94.27)	
Yes	72(1.14)	198(5.73)	
Coronary heart disease, N (%)			< 0.0001
No	4950(98.98)	2780(93.20)	
Yes	64(1.02)	216(6.80)	
Diabetes, N (%)			< 0.0001
No	4263(88.64)	1869(66.70)	
DM	400(5.58)	815(22.43)	
IFG	222(3.66)	194(6.88)	
IGT	129(2.13)	118(3.99)	
Dietary intake of flavonoids (mg/day)			
Daidzein	0.91(0.07)	0.65(0.10)	0.05
Genistein	1.35(0.12)	0.91(0.14)	0.03
Glycitein	0.20(0.02)	0.14(0.02)	0.05
Cyanidin	2.66(0.22)	3.05(0.44)	0.4
Petunidin	1.30(0.13)	1.32(0.16)	0.85
Delphinidin	1.80(0.17)	1.87(0.22)	0.73
Malvidin	5.24(0.41)	5.59(0.48)	0.43
Pelargonidin	1.76(0.16)	1.59(0.17)	0.35
Peonidin	2.62(0.34)	2.31(0.32)	0.54
Catechin	8.11(0.23)	8.73(0.35)	0.07
Epigallocatechin	16.40(0.87)	20.78(2.25)	0.06
Epicatechin	10.51(0.32)	11.33(0.63)	0.25
Epicatechin-3-gallate	10.53(0.55)	13.29(1.38)	0.06
Epigallocatechin-3-gallate	28.55(1.73)	37.12(5.12)	0.11
Theaflavin	1.50(0.08)	1.77(0.10)	0.02
Thearubigins	86.34(4.38)	101.33(5.60)	0.01
Eriodictyol	0.19(0.01)	0.17(0.02)	0.32
Hesperetin	9.04(0.33)	8.71(0.47)	0.55
Naringenin	3.39(0.19)	3.41(0.26)	0.94
Apigenin	0.28(0.06)	0.24(0.02)	0.39
Luteolin	0.78(0.03)	0.75(0.03)	0.42
Isorhamnetin	0.90(0.03)	0.93(0.04)	0.55
Kaempferol	4.87(0.13)	5.20(0.16)	0.06
Myricetin	1.54(0.05)	1.79(0.10)	0.03
Quercetin	11.66(0.27)	12.42(0.30)	0.03
Theaflavin-3,3’-digallate	1.65(0.09)	1.95(0.11)	0.02
Theaflavin-3’-gallate	1.40(0.08)	1.65(0.10)	0.02
Theaflavin 3-gallate	1.19(0.06)	1.40(0.08)	0.02
Gallocatechin	1.66(0.07)	2.02(0.14)	0.02
Subtotal Catechins	75.75(3.63)	93.26(9.73)	0.09
Total Isoflavones	2.46(0.22)	1.70(0.26)	0.03
Total Anthocyanidins	15.39(0.97)	15.73(1.15)	0.76
Total Flavan 3-ols	167.83(7.47)	201.36(12.87)	0.01
Total Flavanones	12.62(0.48)	12.29(0.69)	0.67
Total Flavones	1.06(0.07)	0.98(0.04)	0.29
Total Flavonols	18.97(0.43)	20.34(0.51)	0.02
Total Sum of all 29 flavonoids	218.34(8.07)	252.40(13.05)	0.02

### 3.2 Associations between flavonoid intake and prevalence of hypertension

To assess the potential association between flavonoid intake and risk of hypertension, analyses were performed using weighted logistic regression. The association was fully adjusted for age, race, sex, BMI, daily energy intake, smoking status, alcohol drinking, education, total MET of PA, PIR, hyperlipidemia, heart attack, stroke, congestive heart failure, coronary heart disease, and diabetes. Since more than 50% of the surveyed population reported no intake of isoflavones, we divided isoflavone consumption into two groups based on median intake. The remaining subcategories of flavonoids were categorized into quartiles based on their intake. After fully adjusting for weighted logistic regression (**Table 2**), we found that total anthocyanin intake was inversely associated with the prevalence of hypertension in the fourth quartile [0.81(0.66,0.99), p = 0.04] compared with the first quartile, however, the trend P value was not significant. Similarly, total Flavan-3-ols intake was inversely associated with the prevalence of hypertension in the third quartile [0.79(0.63,0.99), p = 0.04] compared with the first quartile, however, the trend P value was not significant. These results may indicate a nonlinear association between the prevalence of hypertension and the intake of total anthocyanins as well as total flavan-3-ols.

**Table 2:** Associations between flavonoid intake and hypertension.

Flavonoid intake	Q1	Q2		Q3		Q4		
		OR (95% CI)	P Value	OR (95% CI)	P Value	OR (95% CI)	P Value	p for trend
Total Sum of all 29 flavonoids(mg/day)	≤ 27.416	27.416 - 70.402		70.402-239.169		>239.169		
Crude model	ref	1.05(0.83,1.34)	0.66	1.06(0.86,1.30)	0.58	1.00(0.85,1.17)	0.98	0.63
Model 1	ref	0.93(0.71,1.22)	0.61	0.73(0.57,0.93)	0.01	0.78(0.66,0.92)	0.004	0.02
Model 2	ref	1.00(0.76,1.31)	0.99	0.80(0.63,1.02)	0.07	0.85(0.71,1.02)	0.08	0.11
Model 3	ref	0.99(0.74,1.32)	0.93	0.80(0.63,1.02)	0.07	0.85(0.71,1.02)	0.07	0.11
Subtotal Catechins (mg/day)	≤ 5.466	5.466-15.942		15.942-70.718		>70.718		
Crude model	ref	1.03(0.81,1.30)	0.81	0.96(0.78,1.17)	0.67	1.01(0.86,1.20)	0.88	0.86
Model 1	ref	0.91(0.71,1.17)	0.45	0.75(0.59,0.95)	0.02	0.82(0.69,0.98)	0.03	0.17
Model 2	ref	0.96(0.74,1.25)	0.75	0.81(0.63,1.04)	0.09	0.88(0.73,1.07)	0.20	0.45
Model 3	ref	0.96(0.74,1.24)	0.75	0.82(0.63,1.06)	0.13	0.89(0.74,1.07)	0.21	0.47
Total Isoflavones (mg/day)	≤0.01	0.01-210.51						
Crude model	ref	0.78(0.69,0.88)	<0.001					
Model 1	ref	0.87(0.77,1.00)	0.05					
Model 2	ref	0.92(0.80,1.06)	0.27					
Model 3	ref	0.93(0.80,1.08)	0.31					
Total Anthocyanidins (mg/day)	≤0.145	0.145-2.315		2.315-12.804		>12.804		
Crude model	ref	1.02(0.83,1.26)	0.84	1.04(0.83,1.29)	0.74	1.02(0.83,1.25)	0.83	0.93
Model 1	ref	0.91(0.71,1.16)	0.43	0.83(0.65,1.08)	0.16	0.72(0.58,0.88)	0.002	0.004
Model 2	ref	0.93(0.72,1.20)	0.57	0.90(0.69,1.17)	0.40	0.79(0.64,0.98)	0.04	0.04
Model 3	ref	0.92(0.71,1.19)	0.49	0.89(0.68,1.16)	0.37	0.81(0.66,0.99)	0.04	0.08
Total Flavan-3-ols (mg/day)	≤5.587	5.587-16.895		16.895-170.916		>170.916		
Crude model	ref	1.03(0.82,1.30)	0.80	0.97(0.80,1.18)	0.76	0.98(0.83,1.15)	0.78	0.69
Model 1	ref	0.91(0.70,1.19)	0.49	0.71(0.57,0.89)	0.004	0.78(0.66,0.93)	0.01	0.1
Model 2	ref	0.96(0.73,1.26)	0.75	0.77(0.62,0.97)	0.03	0.84(0.70,1.02)	0.08	0.29
Model 3	ref	0.97(0.73,1.28)	0.79	0.79(0.63,0.99)	0.04	0.85(0.70,1.02)	0.08	0.26
Total Flavanones (mg/day)	≤0.07	0.07-0.792		0.792-19.661		>19.661		
Crude model	ref	1.00(0.83,1.21)	0.98	0.89(0.72,1.09)	0.26	1.00(0.81,1.24)	1.00	0.77
Model 1	ref	0.98(0.77,1.26)	0.88	0.81(0.63,1.05)	0.11	0.76(0.58,0.99)	0.05	0.04
Model 2	ref	1.05(0.83,1.33)	0.67	0.89(0.69,1.17)	0.40	0.85(0.65,1.10)	0.20	0.13
Model 3	ref	1.05(0.81,1.36)	0.70	0.91(0.68,1.21)	0.48	0.84(0.63,1.10)	0.19	0.11
Total Flavones (mg/day)	≤0.21	0.21-0.558		0.558-1.144		>1.144		
Crude model	ref	0.99(0.84,1.17)	0.88	0.91(0.75,1.09)	0.28	0.93(0.75,1.14)	0.47	0.44
Model 1	ref	0.84(0.68,1.04)	0.11	0.76(0.62,0.93)	0.01	0.73(0.58,0.91)	0.01	0.01
Model 2	ref	0.87(0.71,1.08)	0.20	0.82(0.66,1.02)	0.08	0.81(0.64,1.01)	0.06	0.12
Model 3	ref	0.89(0.72,1.11)	0.28	0.84(0.66,1.06)	0.13	0.81(0.65,1.02)	0.07	0.12
Total Flavonols (mg/day)	≤7.6	7.6-13.727		13.727-23.549		>23.549		
Crude model	ref	1.02(0.82,1.27)	0.86	0.97(0.78,1.20)	0.77	1.15(0.94,1.39)	0.16	0.09
Model 1	ref	0.86(0.66,1.14)	0.29	0.80(0.63,1.02)	0.07	0.96(0.78,1.20)	0.74	0.78
Model 2	ref	0.90(0.69,1.18)	0.44	0.87(0.68,1.10)	0.23	1.05(0.82,1.34)	0.68	0.29
Model 3	ref	0.91(0.69,1.21)	0.49	0.88(0.68,1.14)	0.31	1.08(0.84,1.39)	0.53	0.2

Crude model: unadjusted. Model 1: adjusted by age, race, sex, BMI. Model 2: adjusted by age, race, sex, BMI, daily energy intake, smoking status, alcohol drinking r, and education. Model 3: adjusted by age, race, sex, BMI, daily energy intake, smoking status, alcohol drinking, education, total MET of PA, PIR, hyperlipidemia, heart attack, stroke, congestive heart failure, coronary heart disease, diabetes.

**Figure 2:**
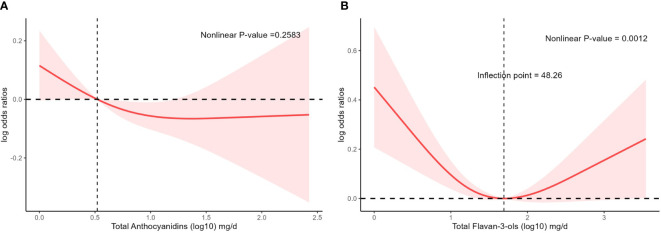
The association of flavonoid intake with prevalence of hypertension by restricted cubic splines. The y-axis stands for the Log odds ratio of hyperlipidemia, and the X-axis stands for the log10 transformed intake of total anthocyanidins **(A)** and total flavan-3-ols **(B)**. Models by restricted cubic splines were adjusted for age, race, sex, BMI, daily energy intake, smoking status, alcohol drinking, education, total MET of PA, PIR, hyperlipidemia, heart attack, stroke, congestive heart failure, coronary heart disease, and diabetes.

To further explore whether there was a nonlinear association between the prevalence of hypertension and intake of total anthocyanins as well as total flavan-3-ols, we conducted analyses using restricted cubic spline bars (**Figure 2**). The results showed that the non-linear relationship between the prevalence of hypertension and intake of total anthocyanins was not significant (p=0.2583, **Figure 2A**). However, there was a significant non-linear relationship between the prevalence of hypertension and intake of total flavan-3-ols (p=0.0012, **Figure 2B**). The relationship between the prevalence of hypertension and intake of total flavan-3-ols showed a U-shaped pattern. We found a significant negative correlation between the prevalence of hypertension and intake of total flavan-3-ols when the intake of total flavan-3-ols was less than 48.26 mg/day.

### 3.3 Subgroup analysis

To identify subgroup effects and explore interaction effects between flavonoid intake and the prevalence of hypertension, analyses were stratified according to age, sex, race, education, BMI, smoking status, alcohol drinking, PIR, daily energy intake, total MET of PA, hyperlipidemia, heart attack, stroke, congestive heart failure, coronary artery disease, and diabetes, and fully adjusted for each stratum by weighted logistic regression.

**Figure 3:**
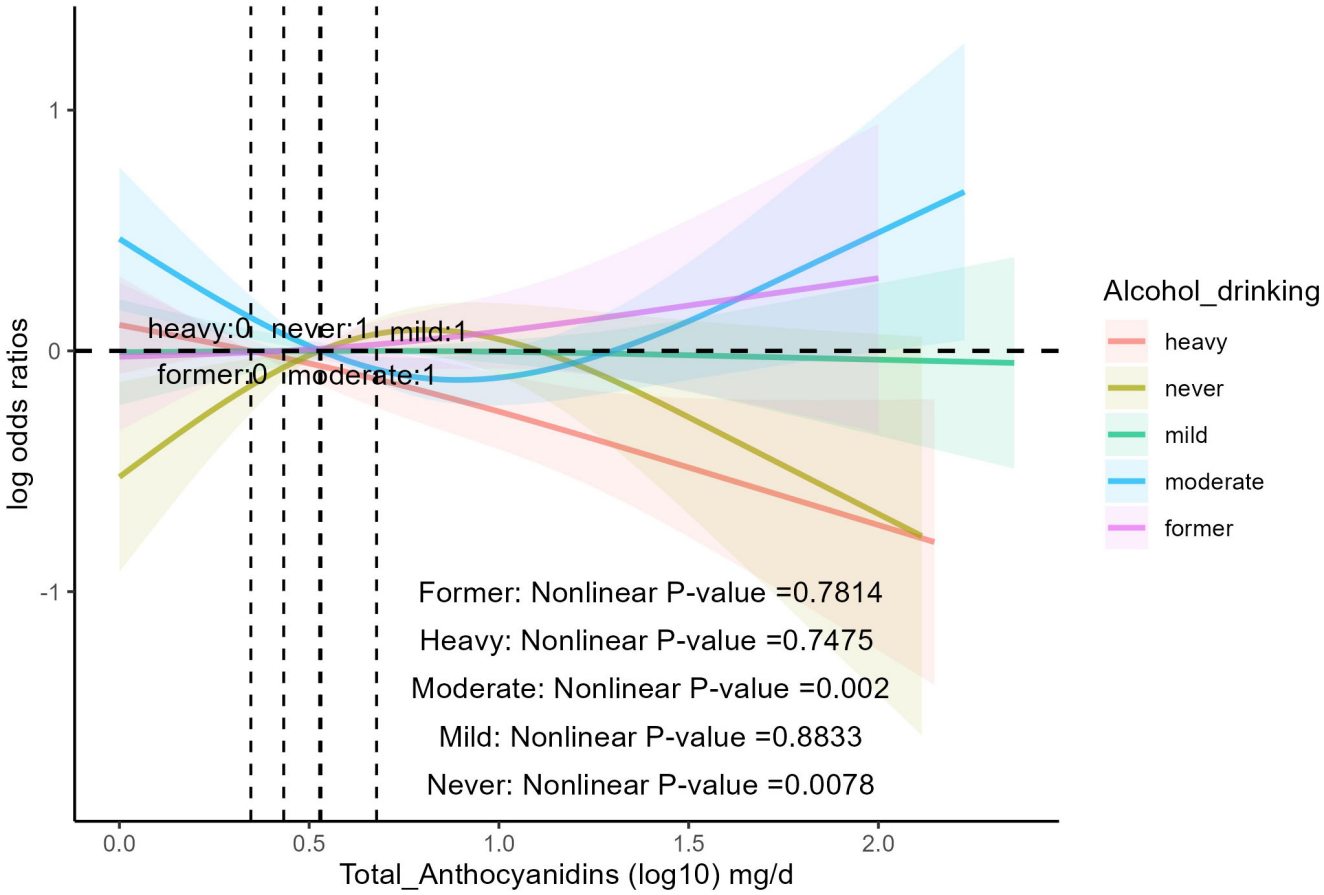
The association of flavonoid intake with the prevalence of hypertension on alcohol drinking by restricted cubic splines. The y-axis stands for the Log odds ratio of hypertension, and the X-axis stands for the log10 transformed intake of total anthocyanidins. Models by restricted cubic splines were adjusted for age, race, sex, BMI, daily energy intake, smoking status, education, total MET of PA, PIR, hyperlipidemia, heart attack, stroke, congestive heart failure, coronary heart disease, and diabetes.

The analysis (**Table 3**) showed that age, sex, race, education, BMI, smoking status, PIR, daily energy intake, total MET of PA, history of hyperlipidemia, heart attack, stroke, congestive heart failure, coronary heart disease, and diabetes did not influence the association between total anthocyanin intake and prevalence of hypertension. However, the relationship between total anthocyanin intake and the prevalence of hypertension was influenced by Alcohol drinking (p for interaction = 0.02). For participants who were heavy drinkers, the fourth quartile of total anthocyanins intake was negatively associated with the prevalence of hypertension compared with the first quartile [0.62(0.40,0.97) p = 0.04], and the trend P value was significant. For participants who were moderate drinkers, the third quartile of total anthocyanin intake was negatively associated with the prevalence of hypertension compared with the first quartile [0.57(0.36,0.89), p = 0.02]. However, the trend p-value was non-significant. We also found a decreasing trend in the prevalence of hypertension with increasing total anthocyanin intake in heavy-drinking participants, unlike the trend in participants with other levels of alcohol consumption. This trend was different from the trend for participants with other levels of alcohol consumption (**Figure 3**). In addition, we found that for participants of all ages, the fourth quartile of total anthocyanins intake was negatively associated with the prevalence of hypertension compared with the first quartile [p = 0.002, p = 0.04, and p < 0.001, respectively], and the trend p-values were all significant. For participants older than 60 years, the second, third, and fourth quartiles of total anthocyanins intake were negatively associated with the prevalence of hypertension compared with the first quartile [p = 0.04, p = 0.03, and p < 0.001, respectively].

**Table 3:** Subgroup analysis between hypertension and total anthocyanidins.

Variables	Q1	Q2		Q3		Q4			
		OR (95%CI)	p Value	OR (95%CI)	p Value	OR (95%CI)	p Value	p for trend	p for interaction
Age									0.65
20-39	ref	0.91(0.66,1.25)	0.54	0.71(0.50,1.02)	0.06	0.56(0.39,0.80)	0.002	0.003	
40-59	ref	0.88(0.58,1.34)	0.55	0.91(0.62,1.36)	0.65	0.70(0.49,1.00)	0.04	0.04	
≥60	ref	0.67(0.45,0.98)	0.04	0.59(0.37,0.94)	0.03	0.49(0.33,0.70)	<0.001	0.002	
Race									0.17
Non-Hispanic White	ref	0.97(0.73,1.28)	0.81	1.06(0.79,1.42)	0.71	1.00(0.76,1.33)	0.97	0.96	
Non-Hispanic Black	ref	1.40(0.99,1.98)	0.05	1.04(0.72,1.50)	0.84	1.42(0.98,2.05)	0.06	0.18	
Mexican American	ref	0.68(0.40,1.17)	0.16	0.75(0.41,1.37)	0.35	0.70(0.40,1.20)	0.19	0.49	
Others	ref	1.70(0.97,2.97)	0.06	1.34(0.89,2.02)	0.15	1.09(0.58,2.06)	0.78	0.45	
Sex									0.97
Female	ref	1.03(0.74,1.41)	0.88	1.02(0.74,1.41)	0.89	1.06(0.79,1.42)	0.69	0.64	
Male	ref	1.04(0.80,1.34)	0.78	1.09(0.83,1.42)	0.53	1.03(0.76,1.40)	0.84	0.98	
Education									0.44
Less than 9th grade	ref	0.57(0.24,1.32)	0.18	0.68(0.36,1.26)	0.21	0.50(0.25,0.99)	0.05	0.1	
9-11th grade (includes 12th grade with no diploma)	ref	1.21(0.80,1.84)	0.36	1.13(0.70,1.81)	0.61	1.21(0.72,2.02)	0.47	0.66	
High School graduate/GED or equivalent	ref	1.26(0.78,2.05)	0.34	1.48(0.98,2.24)	0.06	1.36(0.93,1.99)	0.11	0.24	
Some college or AA degree	ref	0.96(0.71,1.29)	0.79	0.85(0.59,1.23)	0.38	1.15(0.84,1.58)	0.37	0.17	
College graduate or above	ref	0.92(0.57,1.48)	0.73	1.12(0.73,1.73)	0.59	1.05(0.70,1.55)	0.82	0.8	
BMI									0.46
<25	ref	1.19(0.84,1.68)	0.32	1.23(0.77,1.98)	0.38	1.31(0.94,1.82)	0.10	0.25	
25-30	ref	1.17(0.86,1.59)	0.31	1.06(0.77,1.45)	0.72	1.03(0.73,1.46)	0.86	0.77	
>30	ref	0.94(0.70,1.26)	0.66	1.15(0.86,1.55)	0.33	1.33(0.94,1.88)	0.11	0.07	
Smoking status									0.13
Never	ref	1.12(0.85,1.48)	0.40	1.16(0.84,1.61)	0.36	0.99(0.75,1.30)	0.93	0.33	
Former	ref	0.74(0.51,1.08)	0.12	0.63(0.44,0.90)	0.01	0.81(0.55,1.19)	0.27	0.98	
Now	ref	1.04(0.72,1.51)	0.84	1.25(0.82,1.92)	0.29	1.16(0.68,1.97)	0.57	0.58	
Alcohol drinking									0.02
Former	ref	0.84(0.48,1.48)	0.53	1.54(0.74,3.21)	0.23	1.13(0.69,1.87)	0.61	0.37	
Heavy	ref	1.06(0.71,1.58)	0.76	0.73(0.47,1.13)	0.16	0.62(0.40,0.97)	0.04	0.02	
Moderate	ref	1.05(0.68,1.62)	0.82	0.57(0.36,0.89)	0.02	0.97(0.60,1.57)	0.90	0.82	
Mild	ref	1.08(0.75,1.54)	0.67	1.22(0.84,1.77)	0.29	1.17(0.86,1.61)	0.31	0.45	
Never	ref	0.75(0.41,1.35)	0.33	1.23(0.67,2.25)	0.50	0.95(0.49,1.83)	0.87	0.97	
PIR									0.22
<1.5	ref	1.08(0.80,1.46)	0.60	0.90(0.71,1.14)	0.37	0.84(0.60,1.16)	0.28	0.17	
1.5-3.5	ref	1.12(0.79,1.58)	0.52	1.40(0.95,2.07)	0.09	1.31(0.91,1.88)	0.14	0.22	
>3.5	ref	0.92(0.66,1.28)	0.61	0.90(0.64,1.28)	0.55	0.94(0.71,1.24)	0.63	0.92	
Daily energy intake									0.97
<1955	ref	1.04(0.80,1.35)	0.76	1.01(0.76,1.35)	0.93	1.01(0.75,1.35)	0.97	0.93	
≥1955	ref	1.01(0.75,1.36)	0.96	1.07(0.80,1.44)	0.64	1.05(0.79,1.40)	0.72	0.76	
Total MET of PA									0.64
<2400	ref	1.07(0.78,1.47)	0.68	1.14(0.84,1.55)	0.40	1.14(0.86,1.51)	0.35	0.41	
≥2400	ref	0.98(0.75,1.29)	0.90	0.92(0.68,1.25)	0.60	0.90(0.65,1.24)	0.49	0.54	
Hyperlipidemia									0.06
No	ref	1.23(0.83,1.81)	0.30	1.02(0.59,1.76)	0.94	0.75(0.48,1.16)	0.19	0.05	
Yes	ref	0.93(0.73,1.19)	0.57	1.04(0.82,1.31)	0.74	1.12(0.91,1.39)	0.28	0.12	
Heart attack									0.17
No	ref	1.01(0.81,1.27)	0.90	1.02(0.82,1.28)	0.85	1.02(0.83,1.27)	0.82	0.86	
Yes	ref	0.87(0.30,2.55)	0.80	2.64(0.87,7.94)	0.08	0.73(0.30,1.77)	0.47	0.37	
Stroke									0.06
No	ref	1.03(0.83,1.28)	0.78	1.01(0.81,1.27)	0.90	1.02(0.83,1.26)	0.83	0.91	
Yes	ref	0.47(0.15,1.40)	0.17	1.69(0.56,5.06)	0.34	1.63(0.45,5.86)	0.44	0.21	
Congestive heart failure									0.53
No	ref	1.01(0.81,1.26)	0.93	1.04(0.83,1.30)	0.72	1.03(0.84,1.26)	0.77	0.82	
Yes	ref	0.84(0.20, 3.48)	0.80	2.47(0.48,12.78)	0.27	1.61(0.40, 6.42)	0.49	0.43	
Coronary heart disease									0.78
No	ref	1.01(0.81,1.26)	0.92	1.02(0.82,1.26)	0.87	1.00(0.81,1.23)	0.98	0.91	
Yes	ref	1.11(0.37,3.37)	0.85	1.60(0.59,4.33)	0.34	0.93(0.31,2.82)	0.89	0.62	
Diabetes									0.49
No	ref	1.00(0.78,1.27)	0.98	1.04(0.82,1.33)	0.73	1.06(0.87,1.30)	0.53	0.5	
DM	ref	1.28(0.77,2.13)	0.33	1.41(0.87,2.29)	0.16	1.19(0.78,1.82)	0.42	0.92	
IFG	ref	0.70(0.32,1.53)	0.37	0.41(0.15,1.16)	0.09	0.51(0.20,1.30)	0.15	0.29	
IGT	ref	0.81(0.29,2.23)	0.67	0.67(0.29,1.57)	0.34	0.98(0.36,2.65)	0.96	0.72	

The subgroup analyses were adjusted for all covariates except the stratification variable itself.

**Figure 4:**
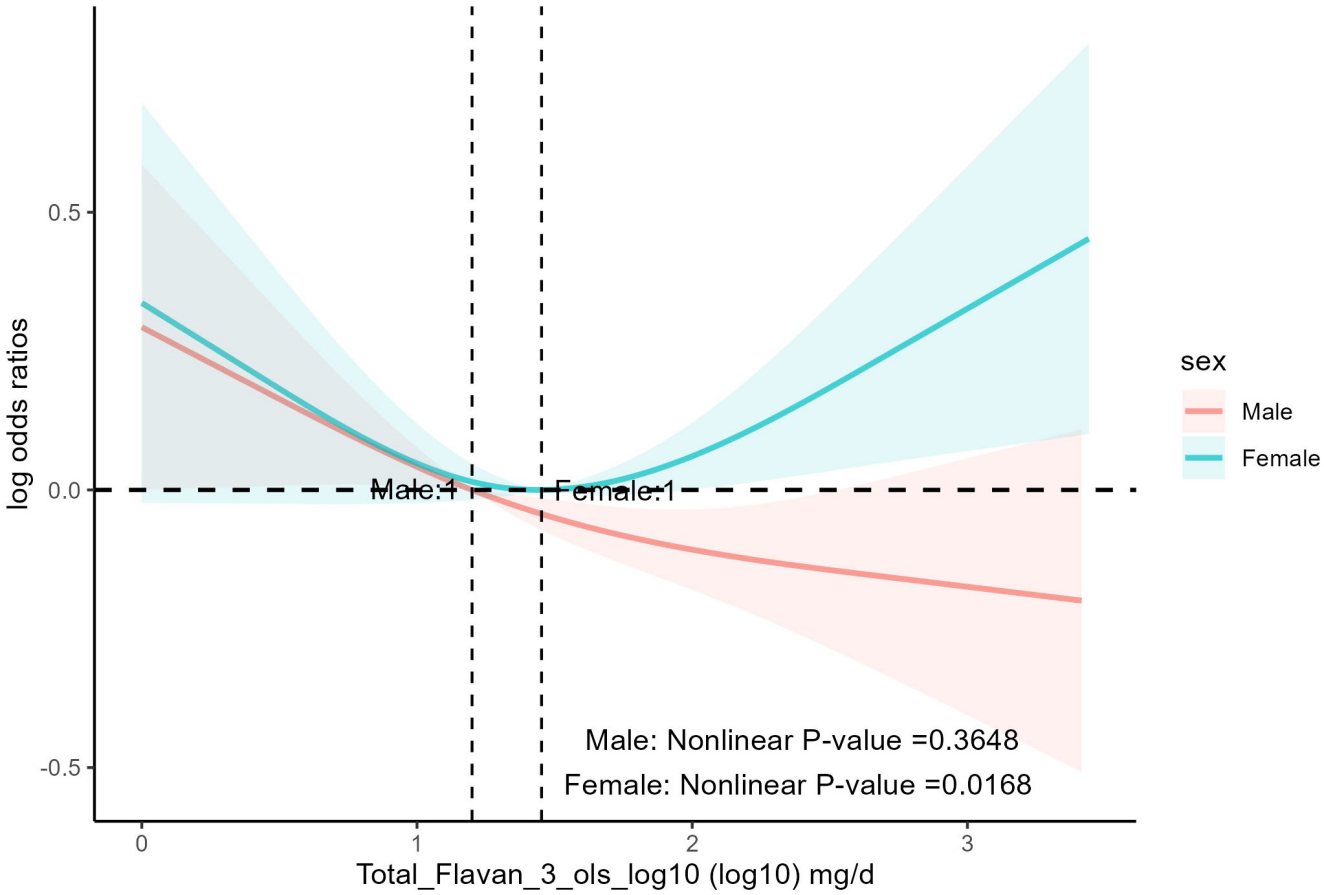
The association of flavonoid intake with prevalence of hypertension on sex by restricted cubic splines. The y-axis stands for the Log odds ratio of hypertension, and the X-axis stands for the log10 transformed intake of total flavan-3-ols. Models by restricted cubic splines were adjusted for age, race, BMI, daily energy intake, smoking status, alcohol drinking, education, total MET of PA, PIR, hyperlipidemia, heart attack, stroke, congestive heart failure, coronary heart disease, and diabetes.

It was found (**Table 4**) that age, race, education, BMI, smoking status, alcohol drinking, PIR, daily energy intake, total MET of PA, history of hyperlipidemia, heart attack, stroke, congestive heart failure, coronary heart disease, and diabetes mellitus did not influence the association between total anthocyanin intake and prevalence of hypertension. However, the relationship between total flavan-3-ols intake and the prevalence of hypertension was influenced by gender (p for interaction = 0.03). As total flavan-3-ols intake increased, the prevalence of hypertension in male participants showed a decreasing trend. Unfortunately, the other three quartiles were not statistically significant compared to the first quartile, and the trend p-value was non-significant (**Table 4**, **Figure 4**). Additionally, we found that for participants aged 20-39 years, the third and fourth quartiles of total flavan-3-ols intake were negatively correlated with the prevalence of hypertension compared to the first quartile [p = 0.002, p = 0.003, respectively], and the trend p-value was significant.

**Table 4:** Subgroup analysis between hypertension and total flavan 3-ols.

Variables	Q1	Q2		Q3		Q4			
		OR (95%CI)	p Value	OR (95%CI)	p Value	OR (95%CI)	p Value	p for trend	p for interaction
Age									0.31
20-39	ref	0.66(0.41,1.07)	0.09	0.53(0.35,0.79)	0.002	0.52(0.34,0.78)	0.003	0.03	
40-59	ref	0.94(0.64,1.40)	0.77	0.80(0.61,1.04)	0.09	0.92(0.70,1.21)	0.56	0.91	
≥60	ref	1.04(0.65,1.66)	0.87	0.73(0.50,1.08)	0.11	0.70(0.42,1.16)	0.16	0.14	
Race									0.98
Non-Hispanic White	ref	1.04(0.77,1.40)	0.80	0.96(0.72,1.27)	0.76	0.98(0.80,1.20)	0.83	0.77	
Non-Hispanic Black	ref	1.04(0.72,1.52)	0.82	1.10(0.81,1.50)	0.52	0.98(0.72,1.33)	0.89	0.71	
Mexican American	ref	1.05(0.62,1.79)	0.85	0.90(0.47,1.71)	0.74	1.11(0.68,1.80)	0.67	0.57	
Others	ref	0.97(0.57,1.66)	0.91	0.99(0.62,1.58)	0.96	0.78(0.50,1.22)	0.28	0.2	
Sex									0.03
Female	ref	0.94(0.66,1.33)	0.71	1.19(0.87,1.64)	0.28	1.17(0.87,1.58)	0.29	0.18	
Male	ref	1.08(0.82,1.43)	0.56	0.83(0.65,1.03)	0.13	0.83(0.65,1.07)	0.15	0.17	
Education									0.88
Less than 9th grade	ref	1.10(0.63,1.92)	0.73	1.34(0.69,2.60)	0.37	1.48(0.72,3.05)	0.28	0.33	
9-11th grade (includes 12th grade with no diploma)	ref	1.17(0.72,1.90)	0.51	0.91(0.58,1.43)	0.67	1.17(0.71,1.94)	0.53	0.57	
High School graduate/GED or equivalent	ref	1.04(0.67,1.62)	0.86	1.11(0.74,1.66)	0.61	1.22(0.85,1.74)	0.27	0.31	
Some college or AA degree	ref	0.98(0.71,1.36)	0.92	1.06(0.73,1.54)	0.76	0.98(0.72,1.34)	0.91	0.8	
College graduate or above	ref	1.20(0.75,1.91)	0.43	1.00(0.66,1.52)	0.99	0.94(0.63,1.40)	0.75	0.25	
BMI									0.7
<25	ref	0.94(0.59,1.49)	0.77	0.96(0.63,1.46)	0.84	0.92(0.64,1.34)	0.67	0.8	
25-30	ref	1.36(0.94,1.98)	0.10	1.28(0.99,1.67)	0.06	1.13(0.77,1.67)	0.53	0.59	
>30	ref	1.06(0.76,1.48)	0.73	0.96(0.69,1.33)	0.80	1.12(0.84,1.51)	0.42	0.35	
Smoking status									0.32
Never	ref	0.94(0.69,1.28)	0.69	1.06(0.78,1.43)	0.70	1.07(0.84,1.35)	0.58	0.44	
Former	ref	1.20(0.79,1.83)	0.37	0.83(0.54,1.26)	0.37	0.84(0.57,1.23)	0.36	0.13	
Now	ref	0.99(0.60,1.66)	0.98	0.83(0.51,1.34)	0.43	0.88(0.57,1.38)	0.58	0.64	
Alcohol drinking									0.45
Former	ref	0.95(0.59,1.52)	0.81	1.18(0.64,2.17)	0.59	1.17(0.67,2.02)	0.57	0.55	
Heavy	ref	1.06(0.66,1.68)	0.82	0.97(0.64,1.49)	0.90	0.67(0.46,0.97)	0.04	0.05	
Moderate	ref	0.78(0.47,1.29)	0.32	0.66(0.40,1.11)	0.11	0.88(0.50,1.54)	0.64	0.81	
Mild	ref	1.14(0.81,1.61)	0.45	0.99(0.75,1.30)	0.95	0.93(0.68,1.28)	0.67	0.41	
Never	ref	1.08(0.57,2.07)	0.81	1.16(0.74,1.82)	0.51	1.51(0.89,2.56)	0.12	0.14	
PIR									0.8
<1.5	ref	0.96(0.68,1.34)	0.80	1.08(0.77,1.53)	0.65	0.91(0.65,1.26)	0.56	0.51	
1.5-3.5	ref	1.04(0.75,1.44)	0.81	0.87(0.66,1.16)	0.34	0.87(0.61,1.23)	0.42	0.45	
>3.5	ref	1.07(0.75,1.52)	0.71	1.01(0.73,1.38)	0.97	1.09(0.83,1.44)	0.52	0.53	
Daily energy intake									0.06
<1955	ref	0.92(0.70,1.19)	0.51	1.09(0.84,1.43)	0.50	1.12(0.89,1.40)	0.33	0.18	
≥1955	ref	1.15(0.86,1.55)	0.33	0.91(0.70,1.17)	0.44	0.89(0.69,1.16)	0.40	0.24	
Total MET of PA									0.62
<2400	ref	0.95(0.70,1.30)	0.76	1.03(0.75,1.43)	0.84	0.95(0.73,1.23)	0.69	0.6	
≥2400	ref	1.10(0.76,1.60)	0.60	0.89(0.67,1.19)	0.42	0.97(0.75,1.27)	0.84	0.76	
Hyperlipidemia									0.06
No	ref	0.83(0.51,1.35)	0.44	0.75(0.53,1.05)	0.09	0.57(0.39,0.84)	0.01	0.02	
Yes	ref	1.11(0.85,1.43)	0.44	1.07(0.84,1.38)	0.58	1.13(0.93,1.37)	0.22	0.31	
Heart attack									0.24
No	ref	1.04(0.82,1.31)	0.74	0.99(0.81,1.22)	0.94	0.96(0.81,1.13)	0.62	0.38	
Yes	ref	1.16(0.45,2.98)	0.75	0.63(0.20,1.99)	0.42	1.88(0.64,5.57)	0.24	0.15	
Stroke									0.36
No	ref	1.02(0.80,1.30)	0.88	0.95(0.78,1.17)	0.65	0.98(0.83,1.16)	0.83	0.86	
Yes	ref	2.10(0.38,11.56)	0.38	2.67(0.72, 9.95)	0.14	1.07(0.33, 3.48)	0.91	0.4	
Congestive heart failure									0.07
No	ref	1.04(0.82,1.31)	0.74	1.00(0.82,1.22)	0.99	1.00(0.84,1.18)	0.97	0.79	
Yes	ref	4.57(0.48,43.18)	0.18	0.42(0.12, 1.40)	0.15	0.56(0.15, 2.03)	0.36	0.29	
Coronary heart disease									0.29
No	ref	1.02(0.80,1.30)	0.85	0.96(0.79,1.18)	0.71	0.94(0.79,1.12)	0.48	0.35	
Yes	ref	0.87(0.32,2.38)	0.78	1.08(0.39,3.03)	0.88	2.12(0.82,5.54)	0.12	0.11	
Diabetes									0.97
No	ref	1.05(0.78,1.41)	0.75	0.97(0.78,1.22)	0.82	0.96(0.77,1.19)	0.68	0.51	
DM	ref	1.15(0.81,1.64)	0.43	1.02(0.54,1.92)	0.96	1.16(0.76,1.76)	0.49	0.62	
IFG	ref	1.07(0.42,2.70)	0.89	0.84(0.45,1.54)	0.56	0.85(0.37,1.97)	0.70	0.68	
IGT	ref	1.19(0.35,3.99)	0.77	1.63(0.56,4.70)	0.35	1.76(0.58,5.28)	0.30	0.43	

The subgroup analyses were adjusted for all covariates except the stratification variable itself.

## 4 Discussion

Hypertension is one of the common chronic diseases (29). Genetics, lifestyle, and environment have a significant impact on the development of hypertension (30). Among these, diet has a significant effect on blood pressure (31), and diets high in salt, saturated fat, trans fat, and added sugar increase the risk of developing hypertension (32). Healthy eating patterns such as the DASH diet and the Mediterranean diet benefit hypertension regulation and reduce cardiovascular risk (33). The DASH diet includes fruits, vegetables, low-fat dairy products, whole grains, poultry, fish, and nuts; and contains smaller amounts of red meat, sweets, and sugar-containing beverages than the typical diet in the United States (34, 35). The DASH diet reduces the incidence of cardiovascular diseases such as coronary heart disease, stroke, and diabetes (34). A meta-analysis showed that the DASH diet reduced systolic blood pressure by 4.2 mmHg and diastolic blood pressure by 2.5 mmHg (36). The Mediterranean diet consists of fish, monounsaturated fats from olive oil, fruits, vegetables, whole grains, legumes/nuts, and moderate alcohol consumption (37). The Mediterranean diet has been found to reduce cardiovascular mortality, cardiovascular outcomes, and the incidence of risk factors such as obesity, hypertension, metabolic syndrome, and dyslipidemia (38). And the Mediterranean-style diet improves systolic blood pressure and arterial stiffness in older adults (39).

Inflammation plays an important role in the development of hypertension (40). The study found that healthy dietary patterns such as the Mediterranean diet and the DASH diet were associated with lower plasma concentrations of pro-inflammatory markers (41). This may suggest that nutritional manipulation can reduce inflammation. Fruits and vegetables are rich in dietary fiber, vitamins, minerals, flavonoids, and other compounds (42). Our study found a negative association between the risk of hypertension and moderate total anthocyanins intake and total flavan-3-ols intake. The mechanism may be through the scavenging of oxygen free radicals, anti-oxidative stress, and inhibition of inflammatory factor activation, thus inhibiting the inflammatory response and exerting a protective effect on blood vessels and lowering blood pressure (43).

Anthocyanins are flavonoid plant compounds widely distributed in fruits, seeds, vegetables, and flowers (44). Foods such as tart cherries, red raspberries, black soybeans, blueberries, sweet cherries, strawberries, and queen garnet plums are rich in anthocyanins (45). Anthocyanins have anti-inflammatory and antioxidant effects (46). Anthocyanins can induce endothelial nitric oxide synthase expression through the Src-ERK1/2-Sp1 signaling pathway in vascular endothelial cells, increase nitric oxide production (47), and reduce ROS produced by endothelial cell activation (48), thereby improving endothelial dysfunction and regulating blood pressure (49). In addition, anthocyanins significantly inhibited the activation of NLRP3 inflammatory vesicles in the paraventricular nucleus of salt-induced hypertensive rats, reduced local inflammation and oxidative stress, and contributed to the reduction of peripheral sympathetic neural activity and lowered blood pressure (50).

Some clinical studies have found anthocyanins in flowers or fruits to have anti-blood pressure effects. A randomized controlled study found no significant difference between a standardized extract of Hibiscus sabdariffa (standardized to 9.6 mg anthocyanins/dose) and captopril in terms of antihypertensive effects and tolerability in patients with mild to moderate hypertension (51). A study found that anthocyanin-rich plum juice reduced ambulatory blood pressure in both young and elderly people (52). A randomized controlled study found that blueberry anthocyanins improved vascular and cognitive function and reduced 24-hour ambulatory systolic blood pressure in healthy older adults (53). Similarly, our study found that quartiles of total anthocyanin intake were negatively associated with the prevalence of hypertension in adults aged 20 years and older. For those aged 60 years or older, the second to fourth quartiles of total anthocyanin intake was negatively associated with the prevalence of hypertension, and the prevalence of hypertension tended to decrease with increasing total anthocyanin intake in participants aged 60 years or older. It has been found that peroxynitrite increases significantly with age. Peroxynitrite is a highly reactive oxidant that scavenges NO, which controls and regulates vascular tone, and with age, vasodilatory dysfunction triggers hypertension (54). Anthocyanins are the most abundant antioxidants in the diet, with antioxidant effects, reducing reactive oxygen species produced by endothelial cell activation and regulating increased nitric oxide production, thus improving endothelial dysfunction and regulating blood pressure (47–49). This may be why anthocyanin intake reduces the prevalence of hypertension in older participants.

It is recognized that high alcohol consumption increases the prevalence of hypertension (55). A Mendelian study found genetic evidence of a causal relationship between alcohol consumption and cardiovascular diseases such as hypertension, coronary heart disease, and myocardial infarction, a relationship with a consistently increasing risk that rises exponentially with increasing intake (56). A Meta-analysis found a positive linear association between alcohol consumption and diastolic blood pressure (57). Our study found that moderate anthocyanin intake reduced the prevalence of hypertension in participants who were heavy and moderate drinkers. It has been found that excessive intake of alcohol leads to oxidative stress in the body, which causes endothelial dysfunction leading to hypertension (58). Anthocyanins can scavenge free radicals and have an anti-oxidative stress effect (59). This may be the reason why moderate anthocyanin intake reduces the prevalence of hypertension for both heavy and moderate drinking participants.

Flavan-3-ols are widely distributed in fruits, vegetables, and plant products such as tea, cocoa, grapes, cranberries, and apples (60, 61). There is growing interest in the beneficial cardiovascular effects of flavan-3-ols (62). It has been found that NF-κB can mediate a variety of inflammatory responses, and activation of the NF-κB signaling pathway induces an increase in the expression of TNF-α and IL-6, which leads to hypertension and contributes to the development of cardiac remodeling (63). Flavan-3-ol, on the other hand, can scavenge free radicals, which helps to reduce the concentration of cellular oxidants and regulate the cellular redox state, and binds to proteins involved in the NF-κB pathway to inhibit the activation of NF-κB, thus improving inflammatory symptoms and exerting protective effects (64). Basic experimental studies have shown that flavan-3-ols reduce systolic blood pressure in spontaneously hypertensive rats (65). The EPIC-Norfolk-based population study found that flavan-3-ol intake was associated with significant reductions in systolic and diastolic blood pressure (66). A study based on the Korean National Health and Nutrition Examination Survey found that flavan-3-ols intake was negatively associated with the risk of hypertension in non-obese men (67). Multiple meta-analyses demonstrated the beneficial effects of flavan-3-ols on cardiometabolic outcomes, with flavan-3-ols significantly improving blood pressure (68–70). Our study found that moderate lavan-3-ols intake was negatively associated with the prevalence of hypertension. Our findings are in agreement with some of the aforementioned studies. In addition, our study found that there was a significant nonlinear relationship (U-shaped) between the prevalence of hypertension and total flavan-3-ols intake. When the intake of total flavan-3-ols was less than 48.26 mg/day, there was a significant negative correlation between the prevalence of hypertension and the intake of total flavan-3-ols.

Our study has several strengths. Our study demonstrated consistent results for curve fitting and segmented linear regression, indicating stable and reliable results. Our subgroup analyses revealed that we found a significant negative association between dietary anthocyanin intake and the prevalence of hypertension in participants aged 60 years or older. However, there are several limitations to our study. This is a cross-sectional study and causal inferences cannot be made, and we look forward to future prospective studies with large samples and different genders. Dietary flavonoid intake was calculated based on 24-hour dietary recall, which may have the effect of recall bias.

## 5 Conclusions

In conclusion, our study found a negative association between the risk of hypertension and moderate total anthocyanins intake and total flavan-3-ols intake. For those over 60 years of age and heavy drinkers, the prevalence of hypertension decreased with increasing total anthocyanin intake. Our study provides evidence for a population-based study of a negative association between dietary flavonoid intake and the prevalence of hypertension, providing valuable information for tailoring nutritional interventions in the management of hypertension.

## Data availability statement

Publicly available datasets were analyzed in this study. This data can be found here: All NHANES data for this study are publicly available and can be found here: https://wwwn.cdc.gov/nchs/nhanes.

## Ethics statement

The studies involving humans were approved by The NCHS Ethics Review Board approved the NHANES study protocol, and each participant signed an informed consent form. The studies were conducted in accordance with the local legislation and institutional requirements. Written informed consent for participation was not required from the participants or the participants’ legal guardians/next of kin in accordance with the national legislation and institutional requirements.

## Author contributions

YW: Conceptualization, Data curation, Formal analysis, Methodology, Writing – original draft. DM: Visualization, Writing – original draft. QS: Conceptualization, Funding acquisition, Writing – review & editing. HX: Conceptualization, Funding acquisition, Supervision, Writing – review & editing.
